# Efficacy and safety of deferasirox doses of >30 mg/kg per d in patients with transfusion-dependent anaemia and iron overload

**DOI:** 10.1111/j.1365-2141.2009.07908.x

**Published:** 2009-09-18

**Authors:** Ali Taher, Maria D Cappellini, Elliott Vichinsky, Renzo Galanello, Antonio Piga, Tomasz Lawniczek, Joan Clark, Dany Habr, John B Porter

**Affiliations:** 1 American University of BeirutBeirut, Lebanon; 2 Università di Milano, Policlinico Foundation IRCCSMilan, Italy; 3 Children’s Hospital and Research CenterOakland, CA, USA; 4 Università di CagliariCagliari, Italy; 5 Università di TorinoTurin, Italy; 6 Novartis Pharma AGBasel, Switzerland; 7 Novartis Pharmaceuticals CorporationEast Hanover, NJ, USA; 8 University College LondonLondon, UK

**Keywords:** deferasirox, efficacy, safety, transfusion-dependent

## Abstract

The highest approved dose of deferasirox is currently 30 mg/kg per d in many countries; however, some patients require escalation above 30 mg/kg per d to achieve their therapeutic goals. This retrospective analysis investigated the efficacy (based on change in serum ferritin levels) and safety of deferasirox >30 mg/kg per d in adult and paediatric patients with transfusion-dependent anaemias, including β-thalassaemia, sickle cell disease and the myelodysplastic syndromes. In total, 264 patients pooled from four clinical trials received doses of >30 mg/kg per d; median exposure to deferasirox >30 mg/kg per d was 36 weeks. In the overall population there was a statistically significant median decrease in serum ferritin of 440 μg/l (*P*< 0·0001) from pre-dose-escalation to the time-of-analysis; significant decreases were also observed in adult and paediatric patients, as well as β-thalassaemia patients. The adverse event profile in patients who received deferasirox doses of >30 mg/kg per d was consistent with previously published data. There was no worsening of renal or liver function following dose escalation. Deferasirox >30 mg/kg per d effectively reduced iron burden to levels lower than those achieved prior to dose escalation in patients with transfusion-dependent anaemias. This has important implications for patients who are heavily transfused and may require higher doses to reduce body iron burden.

Deferasirox (Exjade®) is a once-daily oral iron chelator approved for the treatment of transfusional iron overload in patients with transfusion-dependent anaemia. During a comprehensive series of 1-year core (Cappellini *et al*, 2006; Galanello *et al*, 2006; Piga *et al*, 2006; Vichinsky *et al*, 2007; Porter *et al*, 2008; Taher *et al*, 2009) and ongoing (Porter *et al*, 2007) extension trials, deferasirox was evaluated at starting doses ranging from 5 to 30 mg/kg per d, with maintenance doses of up to 40 mg/kg per d in the extension trials. The efficacy of deferasirox was primarily evaluated based on the assessment of serum ferritin, which remains the most widely used method for evaluating body iron burden. The measurement of serum ferritin is convenient and inexpensive, and serial measurements provide a relatively robust marker of iron burden; significant correlations between changes in serum ferritin and liver iron concentration (LIC) have been identified across various underlying anaemias (Cappellini *et al*, 2006; Porter *et al*, 2008). Current guidelines for assessing iron burden and monitoring the efficacy of chelation therapy recommend the use of serum ferritin (Thalassaemia International Federation, 2008).

Pharmacological studies have shown that the pharmacokinetics of orally administered deferasirox at doses of 10, 20 and 40 mg/kg per d is linear and drug exposure is dose proportional in patients at steady state (Nisbet-Brown *et al*, 2003). In addition, across the clinical trial programme, response to deferasirox was shown to be dependent on dose. A subsequent analysis based on data from a single study highlighted the additional impact of transfusional iron intake on response (Cohen *et al*, 2008). In this analysis 47%, 55% and 75% of patients in the high (>0·5 mg/kg per d), intermediate (0·3–0·5 mg/kg per d) and low (<0·3 mg/kg per d) iron intake categories respectively, achieved a reduction in LIC with 20 mg/kg per d, while the equivalent proportions at a dose of 30 mg/kg per d were 82%, 83% and 96% (Cohen *et al*, 2008). Therefore it is clear that some patients require dose escalation to >30 mg/kg per d in order to achieve their therapeutic goals, i.e. to have a sufficiently rapid decrease of iron burden in heavily iron-overloaded patients, or to successfully prevent iron accumulation in patients with acceptable iron burden levels.

However, when deferasirox was first licensed in 2005, there was a comparatively small clinical database and a limited number of patients had been exposed to doses of >30 mg/kg per d. As such, doses above 30 mg/kg per d were not recommended in the prescribing information for deferasirox when the drug was first approved. As a larger number of patients have now been exposed to deferasirox doses of >30 mg/kg per d for a prolonged period of time, a retrospective analysis has been performed to investigate the efficacy and safety of such doses. Data for this analysis have been pooled from four prospective clinical trials in patients with various transfusion-dependent anaemias, including β-thalassaemia, sickle cell disease (SCD) and the myelodysplastic syndromes (MDS).

## Methods

### Eligibility criteria

Male or female patients aged ≥2 years with transfusional iron overload, as defined by a LIC of ≥2 mg Fe/g dry weight (dw) at the start of deferasirox treatment, were eligible for inclusion in this analysis. All patients were required to have baseline serum creatinine levels below the upper limit of normal (ULN) and were excluded if alanine aminotransferase (ALT) levels were >250 U/l in the previous year. More detailed enrolment criteria for the four studies have been published elsewhere (Cappellini *et al*, 2006; Vichinsky *et al*, 2007; Porter *et al*, 2008; Taher *et al*, 2009).

Institutional Review Board or Ethics Committee approval was obtained at each participating institution. All patients (or parents/guardians) provided written informed consent. All studies were conducted in accordance with Good Clinical Practice guidelines and the Declaration of Helsinki and were closely monitored by Novartis personnel or a contract organization for compliance to the protocols and the procedures described in them.

### Study design and dosing

This pooled analysis was performed using data from the core and ongoing extension phases of four prospective multicentre, open-label trials in patients who received deferasirox >30 mg/kg per d (Cappellini *et al*, 2006; Vichinsky *et al*, 2007; Porter *et al*, 2008; Taher *et al*, 2009); a summary of these trials is provided in Table I. All four trials used comparable efficacy and safety monitoring and assessment methods and are therefore suitable for pooled analysis.

**Table I:** Summary of trials included in this analysis.

Novartis trial number	Design	Patient population	Patients exposed to deferasirox	Patients contributing to this analysis, *n* (%)
CICL670A0107E (*Study 1*) Cappellini *et al* (2006)	Randomized, comparative deferasirox *versus* DFO (1 year), followed by deferasirox only (4 years)	Adult and paediatric patients with β-thalassaemia major	555	99 (17·8)
CICL670A0108E (*Study 2*) Porter *et al* (2008)	Single arm of deferasirox, non-comparative	Adult and paediatric patients with MDS, DBA and other anaemias of diverse aetiologies	184	34 (18·5)
CICL670A0109E (*Study 3*) Vichinsky *et al* (2007)	Randomized, comparative deferasirox *versus* DFO (1 year), followed by deferasirox only (4 years)	Adult and paediatric patients with SCD	185	32 (17·3)
CICL670A02402E (*Study 4*) Taher *et al* (2009)	Single arm of deferasirox, non-comparative	Adult and paediatric patients with β-thalassaemia major	237*	99 (41·8)

DFO, deferoxamine; MDS, myelodysplastic syndromes; DBA, Diamond-Blackfan anaemia; SCD, sickle cell disease.

* Fifteen patients from a single site were excluded from the final analysis because routine monitoring of study documents at the site could not confirm the accuracy of the data reported (Taher *et al*, 2009).

Initial deferasirox doses in studies 1–3 were 5–30 mg/kg per d, depending on baseline LIC; doses above 30 mg/kg per d were not permitted (Cappellini *et al*, 2006; Vichinsky *et al*, 2007; Porter *et al*, 2008). In the extension studies, investigators were allowed to increase the doses above 30 mg/kg per d up to 40 mg/kg per d in patients in whom the control of body iron was judged inadequate after review/approval by the Safety Monitoring Committee. Dose increases in steps of 5–10 mg/kg per d were recommended after a minimum of 3 months’ treatment for patients with baseline serum ferritin of >500 μg/l who had an increasing serum ferritin trend, or for those with baseline serum ferritin of >1000 μg/l who did not have a serum ferritin decrease.

During the 1-year core phase of study 4, all patients started on deferasirox 20 mg/kg per d except three who started on 10 mg/kg per d (Taher *et al*, 2009). Doses of >30 mg/kg per d were not initially recommended in the extension study, although this was subsequently changed following a protocol amendment. Dose increases of 5–10 mg/kg per d were recommended for patients who had confirmed rises in serum ferritin of ≥1000 μg/l above baseline, or for patients whose serum ferritin remained at >2500 μg/l without showing a downward trend.

### Assessments

Efficacy was assessed based on the monthly serum ferritin observations before and after escalation to doses of >30 mg/kg per d.

Safety was evaluated throughout treatment after escalation to doses of >30 mg/kg per d, based on the incidence and type of adverse events (AEs) and serious AEs, and routine laboratory testing including serum creatinine and liver enzyme levels.

### Patient populations and statistical methods

To evaluate patients who were intended to receive deferasirox >30 mg/kg per d and to avoid variability based on tablet strength (as some patients may have unintentionally received doses of >30 mg/kg per d due to the fact that the only tablet strengths available are 125, 250 or 500 mg), only patients with a prescribed dose of >30 mg/kg per d and an actual daily dose of ≥32·5 mg/kg per d were analysed. The term ‘doses of >30 mg/kg per d’ used in the subsequent text is defined based on these two criteria.

All efficacy analyses are based on patients treated with doses of >30 mg/kg per d, as defined previously, and who had serum ferritin assessments at study baseline and at least once after escalation to doses of >30 mg/kg per d. All safety analyses are based on patients treated with doses of >30 mg/kg per d who had at least one safety assessment performed after escalation to doses of >30 mg/kg per d. Transfusional iron intake was calculated according to the method previously described by Cohen *et al* (2008).

**Fig 1:**
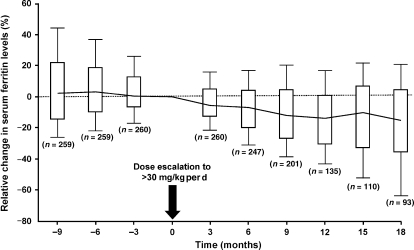
Relative change (%) in serum ferritin levels from pre-dose escalation (efficacy population). Note: The boxes represent the 25th and 75th percentiles, while the whiskers correspond to the 10th and 90th percentiles. The medians are connected.

**Fig 2:**
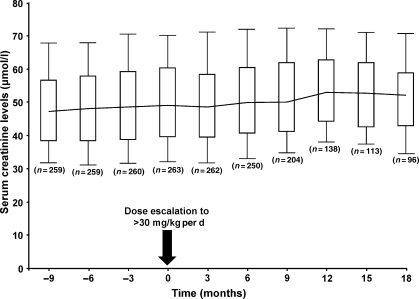
Serum creatinine levels before and after dose escalation to doses >30 mg/kg per d (safety population). Note: The boxes represent the 25th and 75th percentiles, while the whiskers correspond to the 10th and 90th percentiles. The medians are connected.

Laboratory changes over time were summarized by averaging the observed values over 3-month periods. The reference point prior to dose escalation was calculated by averaging the observed values over the previous 3 months. Data were mainly summarized descriptively. Graphical representations were also used to follow change over time; the boxes in Figs 1 and 2 display the 10th and the 90th percentiles and the medians are connected. The last observed serum ferritin value was compared to pre-dose escalation values using paired Wilcoxon tests when the number of patients with both a pre-dose escalation and post-dose escalation value was >10.

## Results

### Patient characteristics

Of 1176 patients who received deferasirox across the four studies, 264 (22·4%) received doses of >30 mg/kg per d. Most of these 264 patients had their doses escalated due to inadequate control of serum ferritin levels. This population was composed primarily of patients with β-thalassaemia (Table II). Median serum ferritin levels at pre-dose escalation in paediatric and adult patients were 3843 and 3930 μg/l, respectively.

**Table II:** Patient characteristics prior to dose escalation.

Variable	>30 mg/kg per d (*n* = 264)
Mean age ± SD, years	19·0 ± 8·4
Median (range)	17·7 (5–53)
Age, *n* (%)	
<16 years	113 (42·8)
≥16 years	151 (57·2)
Male:female	129:135
Race, *n* (%)	
Caucasian	138 (52·3)
Black	28 (10·6)
Oriental	56 (21·2)
Other	42 (15·9)
Underlying anaemia, *n* (%)	
β-thalassaemia	225 (85·2)
MDS	–
DBA/rare anaemias	7 (2·7)
SCD	32 (12·1)
Median serum ferritin (range), μg/l	
Start of deferasirox treatment	3937 (342–25 008)
Pre-dose escalation	3880 (876–15 747)

MDS, myelodysplastic syndromes; DBA, Diamond-Blackfan anaemia; SCD, sickle cell disease.

### Transfusional iron intake

For the 264 patients who had their doses escalated to >30 mg/kg per d, overall median transfusional iron intake was 0·34 mg/kg per d for the 6 months preceding dose escalation, which remained constant once doses had been escalated.

### Deferasirox dosing

After dose escalation, 33 patients (12·5%) received an average deferasirox dose of <32·5 mg/kg per d, 98 patients (37·1%) an average dose of ≥32·5 to <37·5 mg/kg per d, 130 patients (49·2%) an average dose of ≥37·5 to <42·5 mg/kg per d and three patients (1·1%) an average dose of ≥42·5 mg/kg per d. Overall median exposure to deferasirox was 169 weeks, while median exposure from the first to last administration of deferasirox >30 mg/kg per d was 36 weeks. The median of the last dose prior to escalation was 30·0 mg/kg per d, while the median dose after escalation was 37·5 mg/kg per d. Twenty-six patients (9·8%) had 32 dose reductions, primarily decreases according to the study protocol (e.g. body weight change), while 101 patients (38·2%) had drug interruptions, mostly because of missed doses. The median duration of drug interruption was 2 d (range 1–152).

### Change in serum ferritin levels

Overall, 261 patients (98·9%) met the requirements for the efficacy analysis. Pre-escalation serum ferritin levels ranged from *c.*3500 to 4500 μg/l across all underlying anaemias (Table III). In this efficacy population, the median relative change in serum ferritin at the time-of-analysis was −12·8% compared with the level preceding dose escalation to >30 mg/kg per d (Fig 1; Table III). In these patients there was a statistically significant median decrease in serum ferritin of 440 μg/l (*P*< 0·0001; Table III) from pre-dose-escalation to the time-of-analysis.

**Table III:** Median of relative and absolute change in serum ferritin at last observed assessment following dose escalation to >30 mg/kg per d, by subgroup (efficacy population).

Population	n*	Pre-escalation serum ferritin (range), μg/l	Relative change from pre-escalation, %	Absolute change from pre-escalation, μg/l [95% CI]	*P*-value†
All patients	261	3880 (876–15 747)	−12·8	−440 [−618, −286]	<0·0001
β-thalassaemia	222	3794 (876–15 747)	−14·2	−487 [−624, −286]	<0·0001
SCD	32	4516 (1355–11 288)	−5·8	−193 [−724, 259]	ns
DBA/rare anaemias	7	3469 (1141–12 290)	−27·9	−923 [−1610, 579]	–
Adults	150	3930 (1141–15 747)	−11·6	−368 [−568, −160]	0·001
Paediatrics	111	3843 (876–8511)	−15·2	−617 [−839, −289]	<0·0001

DBA, Diamond-Blackfan anaemia; SCD, sickle cell disease; CI, confidence intervals; ns, non-significant.

* Patients with available pre- and post-escalation serum ferritin levels.

† *P*-value based on paired Wilcoxon test, absolute serum ferritin change *versus* pre-escalation.

In patients with β-thalassaemia, deferasirox doses of >30 mg/kg per d significantly reduced serum ferritin at the last observation time to below the levels prior to dose escalation by 487 μg/l (*P*< 0·0001; Table III). In patients with SCD, median serum ferritin levels decreased by 193 μg/l. In paediatric patients (aged <16 years), dose escalation to >30 mg/kg per d significantly reduced serum ferritin levels by 617 μg/l compared to the levels prior to dose escalation (*P*< 0·0001, *n*= 111; Table III). Similarly, doses of >30 mg/kg per d also provided significant reductions in serum ferritin levels by 368 μg/l in adult patients (*P*= 0·001, *n* = 150). There was no significant difference between paediatric and adult patients with respect to the relative median change in serum ferritin from pre-escalation to last observed assessment (−15·2 vs. −11·6; *P*= 0·1984).

### Safety and tolerability

Forty-nine (18·6%) patients had AEs leading to either dose reduction or drug interruption (45 patients had drug interruption, three patients had dose reduction and one patient had both). Median dose prior to interruption was 37·4 mg/kg per d, while median dose prior to reduction was 35 mg/kg per d. Thirty-two of these 49 patients continued treatment with deferasirox until the completion of the study. Of the remaining 17 patients, nine patients still were treated with deferasirox at the cut-off date of this pooled analysis, three patients discontinued the study due to unsatisfactory therapeutic effect as assessed by the investigator, two patients discontinued due to AEs (see section below), two patients withdrew consent, and one patient was lost to follow-up.

#### Study discontinuations

At the time of analysis 32 patients (12·1%) were still on study, while 185 (70·1%) had completed the extension studies. Forty-seven dose-escalated patients (17·8%) permanently discontinued due to: insufficient change in serum ferritin as assessed by the investigator (*n* = 19), AEs (*n* = 10), consent withdrawal (*n*= 10), lost to follow-up (*n*= 4), abnormal laboratory values (*n*= 3) and administrative problems (*n*= 1). The AEs leading to discontinuation were increased ALT, lenticular opacities (classed as a serious AE), increased serum ferritin, cardiac failure in two patients (serious AEs), abdominal discomfort, hypotension (serious AE), dyspnoea (serious AE), gastrointestinal haemorrhage (serious AE) and marrow transplantation due to β-thalassaemia (serious AE). Four of the 10 AEs resulting in discontinuation (lenticular opacities, increased serum ferritin, abdominal discomfort, gastrointestinal haemorrhage) were suspected by the investigators to be drug-related. Median dose prior to discontinuation was 39·5 mg/kg per d; the median time between study discontinuation and the last dose was 1 d (range −1 to 95).

#### Adverse events

Overall, 237 (89·8%) and 173 (65·5%) patients experienced an AE before and after escalation to >30 mg/kg per d, respectively. Most AEs were mild to moderate in nature. Ten patients (3·8%) only had AEs after dose escalation, three of whom received doses >30 mg/kg per d at baseline; nine had baseline serum ferritin levels >1000 μg/l.

Forty-three patients (16·3%) had a drug-related (investigator-assessed) AE after dose escalation to >30 mg/kg per d. The most common drug-related AEs before and after escalation are shown in Table IV. Of the eight patients who had an ALT increase after dose escalation, two had the same AE before dose escalation and four had elevated ALT before receiving deferasirox. None of the three patients who had a serum creatinine increase after dose escalation had this AE before escalation.

**Table IV:** Number (%) of patients with drug-related adverse events as assessed by investigators (observed in >1 patient after dose escalation to >30 mg/kg per d).

	Frequency, *n* (%)	
AE	Before dose escalation	After dose escalation
Total exposure (patient years)	631·6	273·4
ALT increase*	13 (4·9)	8 (3·0)
Vomiting	19 (7·2)	7 (2·7)
Nausea	26 (9·8)	5 (1·9)
Abdominal pain	17 (6·4)	4 (1·5)
Upper abdominal pain	4 (1·5)	4 (1·5)
Blood creatinine increase*	22 (8·3)	3 (1·1)
Abdominal discomfort	2 (0·8)	2 (0·8)
Diarrhoea	16 (6·1)	2 (0·8)
AST increase*	6 (2·3)	2 (0·8)
Transaminase increase*	5 (1·9)	2 (0·8)

ALT, alanine aminotransferase; AST, aspartate aminotransferase.

* Reported as an AE by the investigator.

Forty-two patients (15·9%) had a serious AE following escalation to >30 mg/kg per d; those assessed as possibly drug-related were lenticular opacities, increased transaminases, gastrointestinal haemorrhage, abdominal pain and abnormal liver function tests (*n*= 1 for each). After escalation there were no cases of pancreatitis or obstructive jaundice, one (0·4%) case of gastritis, one (0·4%) case of cholecystitis and five (1·9%) cases of cholelithiasis; no cases of gastritis, cholecystitis or cholelithiasis were considered to be drug-related. There were no deaths among patients who received doses of >30 mg/kg per d.

#### Renal parameters

After escalation to >30 mg/kg per d, the median of relative change in serum creatinine remained unchanged at the end-of-study compared with pre-escalation (Fig 2). Two patients (0·8%) had serum creatinine above the ULN, at two consecutive assessments more than 7 d apart after dose escalation. One of them had normal serum creatinine levels prior to dose escalation. One patient had a urine protein:creatinine (UPUC) ratio >1·0 mg/mg at two consecutive visits after escalation to >30 mg/kg per d. Eight patients (3·0%) had a UPUC ratio >1·0 mg/mg at one visit, seven of whom had a normal UPUC ratio prior to dose escalation. Twenty-seven patients (10·2%) had one episode of abnormal UPUC ratio <1·0 mg/mg, 18 of whom had a normal ratio prior to dose escalation.

#### Hepatic safety

Nine patients (3·4%) had ALT levels of >5 × ULN but <10 × ULN at two consecutive assessments at least 7 d apart, all of whom had ALT >ULN prior to escalation to doses >30 mg/kg per d. Three patients, two of whom who had ALT < ULN prior to dose escalation, had ALT values of >10 × ULN at two consecutive visits after escalation.

#### Auditory and ophthalmic assessments

One patient, who permanently discontinued treatment, had a lenticular opacity assessed as drug-related after escalation to doses of >30 mg/kg per d (39 mg/kg per d); this patient had a normal eye examination at baseline. Six additional patients had ocular abnormalities suggestive of impaired vision (four patients with blurred vision and one each with reduced visual acuity and papilloedema) reported after dose escalation; none were assessed as drug related by the investigator. In 2/7 patients who had an ocular AE of interest, ophthalmological examination revealed normal (*n* = 1) or clinically insignificant (*n* = 1) results at the onset of the AE. The lenticular opacity experienced by one patient was detected by ophthalmologic examination. In two of the remaining four patients, the AEs were preceded and followed by normal ophthalmological examinations. Ophthalmological examinations were not available in the remaining two patients. Four patients reported some hearing loss after dose escalation to >30 mg/kg per d, although none discontinued and one already had hearing loss on lower deferasirox doses. One of these episodes was assessed as drug related.

## Discussion

Previous studies have indicated that some patients require deferasirox doses of >30 mg/kg per d in order to achieve their therapeutic goals. Here we report for the first time that such doses are effective with a comparable safety profile to doses of <30 mg/kg per d. The patients who were escalated to doses of >30 mg/kg per d presented with very high median serum ferritin levels prior to escalation (3880 μg/l overall); 208 patients (78·8%) had levels >2500 μg/l, which has been associated with an increased risk of iron overload-related complications (Olivieri *et al*, 1994; Olivieri & Brittenham, 1997) and shown to require intensification of chelation therapy (Thalassaemia International Federation, 2008).

Despite limited exposure (median 36 weeks), this analysis demonstrates that escalation of deferasirox to doses of >30 mg/kg per d effectively decreased serum ferritin compared with levels prior to dose escalation in patients whose serum ferritin levels were not adequately controlled before escalation. The serum ferritin decreases were significant in the overall population, in adult and paediatric patients, as well as patients with β-thalassaemia. Although patients with SCD and other anaemias were included in this analysis, the patient numbers were too low to provide any meaningful information. This may suggest that iron overload in these populations was sufficiently well managed on deferasirox doses of ≤30 mg/kg per d and they generally did not require escalation to >30 mg/kg per d. However, more than half of the SCD patients (*n* = 18, 56%) had drug interruptions (≥4 in 78% of patients) after dose escalation, which may have impacted on the serum ferritin response. It is also worth noting that the measurement of serum ferritin levels is a less reliable marker of iron overload in SCD than in other anaemias. This is because ferritin is an acute phase reactant therefore levels can be greatly influenced by inflammation (Brownell *et al*, 1986), which is an important factor in the pathophysiology of SCD.

Patients who required escalation to doses of >30 mg/kg per d were heavily iron overloaded, which was reflected by high serum ferritin levels at baseline. These patients also received an intermediate amount of transfusional iron intake (7–14 ml/kg per month) after dose escalation. This emphasizes the need to dose deferasirox based on goal of therapy, iron burden and transfusional iron intake, and highlights that dose escalation may be required in patients with severe iron overload who do not respond sufficiently to doses up to 30 mg/kg per d.

The safety profile observed in patients who received deferasirox doses of >30 mg/kg per d was consistent with that observed in the 1-year core trials (Cappellini *et al*, 2006; Galanello *et al*, 2006; Piga *et al*, 2006; Vichinsky *et al*, 2007; Porter *et al*, 2008). Importantly, there were no AEs observed following escalation to >30 mg/kg per d that were not observed at lower doses before escalation. Serum creatinine levels remained unchanged at the end of study period compared with the pre-escalation level, which suggests that the risk of impaired renal function is low while administering doses of >30 mg/kg per d in heavily iron-overloaded patients. Forty-two patients experienced a serious AE following dose escalation to >30 mg/kg per d, which was consistent with those observed prior to dose escalation and in the 1-year core trials (Cappellini *et al*, 2006; Galanello *et al*, 2006; Piga *et al*, 2006; Vichinsky *et al*, 2007; Porter *et al*, 2008; Taher *et al*, 2009) and typical of those expected in the target population. The safety of doses >30 mg/kg per d was not assessed in patients with serum ferritin levels ≤1000 μg/l as only one patient had such low levels at dose escalation. It is therefore not possible to comment on the safety of doses >30 mg/kg per d in patients with low levels of iron loading. However, in general, high doses should be reserved for patients with very high levels of iron loading who are not responding to doses <30 mg/kg per d.

Limitations of this study include the fact that it was a retrospective analysis and patients who were included were likely to be those who tolerated doses <30 mg/kg per d, which is important to consider when reviewing the safety data.

In conclusion, these findings indicate that escalation of deferasirox doses to >30 mg/kg per d effectively reduces iron burden to lower levels than those achieved prior to dose escalation, without an increase in the incidence of AEs or a worsening of renal or liver function. Deferasirox dose should be titrated to meet individual patient needs as dictated by transfusional iron intake and the goal of therapy (reduction or maintenance of body iron stores). Before considering dose escalation to >30 mg/kg per d to reduce body iron burden in patients with severe iron overload, it is important to assess both the response to therapy (serum ferritin monitoring) and compliance at doses ≤30 mg/kg per d. The dose-escalated patients should be regularly monitored and, once body iron stores fall into an acceptable range, deferasirox dose should be decreased to an appropriate maintenance level.
